# Equal Maintenance of Anti-SARS-CoV-2 Antibody Levels Induced by Heterologous and Homologous Regimens of the BNT162b2, ChAdOx1, CoronaVac and Ad26.COV2.S Vaccines: A Longitudinal Study Up to the 4th Dose of Booster

**DOI:** 10.3390/vaccines12070792

**Published:** 2024-07-18

**Authors:** Tatiana A. do Nascimento, Patricia Y. Nogami, Camille F. de Oliveira, Walter F. F. Neto, Carla P. da Silva, Ana Claudia S. Ribeiro, Alana W. de Sousa, Maria N. O. Freitas, Jannifer O. Chiang, Franko A. Silva, Liliane L. das Chagas, Valéria L. Carvalho, Raimunda S. S. Azevedo, Pedro F. C. Vasconcelos, Igor B. Costa, Iran B. Costa, Luana S. Barbagelata, Wanderley D. das Chagas Junior, Edvaldo T. da Penha Junior, Luana S. Soares, Giselle M. R. Viana, Alberto A. Amarilla, Naphak Modhiran, Daniel Watterson, Lívia M. N. Casseb, Lívia C. Martins, Daniele F. Henriques

**Affiliations:** 1 Department of Arbovirology and Hemorrhagic Fevers, Evandro Chagas Institute, Ananindeua 67030-000, Pará, Brazilanaribeiro@iec.gov.br (A.C.S.R.);; 2 Graduate Program in Virology, Evandro Chagas Institute, Ananindeua 67030-000, Pará, Brazil; 3 Department of Virology, Evandro Chagas Institute, Ananindeua 67030-000, Pará, Brazil; 4 Department of Biological and Health Sciences, University of Pará State, Belém 66087-670, Pará, Brazil; 5 Malaria Basic Research Laboratory, Parasitology Section, Evandro Chagas Institute, Health Surveillance Secretariat, Brazilian Ministry of Health, Ananindeua 67000-000, Pará, Brazil; 6 School of Chemistry and Molecular Biosciences, The University of Queensland, St Lucia, QLD 4072, Australia; 7 The Australian Institute for Biotechnology and Nanotechnology, The University of Queensland, St Lucia, QLD 4072, Australia; 8 Australian Infectious Disease Research Centre, The University of Queensland, St Lucia, QLD 4072, Australia

**Keywords:** COVID-19, neutralizing antibodies, anti-RBD antibodies, vaccine effectiveness, study longitudinal, ChAdOx1-S, BNT162b2, CoronaVac, dose booster

## Abstract

Several technological approaches have been used to develop vaccines against COVID-19, including those based on inactivated viruses, viral vectors, and mRNA. This study aimed to monitor the maintenance of anti-SARS-CoV-2 antibodies in individuals from Brazil according to the primary vaccination regimen, as follows: BNT162b2 (group 1; 22) and ChAdOx1 (group 2; 18). Everyone received BNT162b2 in the first booster while in the second booster CoronaVac, Ad26.COV2.S, or BNT162b2. Blood samples were collected from 2021 to 2023 to analyze specific RBD (ELISA) and neutralizing antibodies (PRNT50). We observed a progressive increase in anti-RBD and neutralizing antibodies in each subsequent dose, remaining at high titers until the end of follow-up. Group 1 had higher anti-RBD antibody titers than group 2 after beginning the primary regimen, with significant differences after the 2nd and 3rd doses. Group 2 showed a more expressive increase after the first booster with BNT162B2 (heterologous booster). Group 2 also presented high levels of neutralizing antibodies against the Gamma and Delta variants until five months after the second booster. In conclusion, the circulating levels of anti-RBD and neutralizing antibodies against the two variants of SARS-CoV-2 were durable even five months after the 4th dose, suggesting that periodic booster vaccinations (homologous or heterologous) induced long-lasting immunity.

## 1. Introduction

COVID-19 is a disease associated with a viral infection caused by SARS-CoV-2, which spread rapidly across the world. The pandemic caused by this new coronavirus became a global public health problem. COVID-19 cases present a broad clinical spectrum, from asymptomatic cases to mild and severe symptoms such as respiratory failure and death. The disease’s severity appears to be associated with age and pre-existing comorbidities [1,2,3].

The SARS-CoV-2 viral particle has a spherical shape of approximately 120 nanometers in diameter. The genome is composed of a single-stranded RNA of positive polarity (+ssRNA), presenting the following four structural proteins: spike (S), envelope (E), membrane (M), and nucleocapsid (N) [4,5]. The S protein is directly linked to the tropism of the virus through the attachment of the virus to the host cell, which happens when the receptor-binding domain (RBD) particle binds to angiotensin-converting enzyme 2 (ACE2) to enter the cell [6].

Given the importance of the RBD region, several studies were directed toward the standardization and validation of serological assays using this target, aimed at epidemiological applications and the detection of neutralizing antibodies, with the latter helping to verify the natural immune protection against infection in individuals, as well as the verification of the effectiveness of the newly developed vaccines [7,8].

The high replication rate of SARS-CoV-2 contributes to the emergence of new variants with high-effective mutations, which modulates the adaptation capacity and increases the transmission efficiency and pathogenicity of the virus. As a result, a worse prognosis is expected, affecting the established clinical measures to combat the pathogen [9,10,11].

In this scenario, several measures have been adopted to fight and control infection by SARS-CoV-2, with the development of vaccines being one of the most sought-after objectives in the world. This type of immunization has been proven to represent the most effective means of controlling and interrupting the spread of the disease [12]. There are currently nine vaccines approved and validated for use by the World Health Organization (WHO), which are administered in two doses at an interval of three weeks and an additional dose (i.e., booster) after six months, as follows: (1) Pfizer/BioNTech Comirnaty (mRNA); (2) IBS/COVISHIELD (vector—inactivated adenovirus); (3) AstraZeneca/AZD1222 (vector—inactivated adenovirus); (4) Janssen/Ad26.COV2 (inactivated viral vector); (5) Moderna COVID-19 (mRNA 1273); (6) Sinopham COVID-19 (inactivated virus); (7) Sinovac-CoronaVac (inactivated virus); (8) Bharat Biotech BBV152 COVAXIN (inactivated virus); (9) Bivalent [13,14,15].

Clinical studies show that the severity of symptoms was significantly lower in people who received the booster dose than those vaccinated only with the primary scheme or those not vaccinated [16]. However, the protection provided by the vaccine decreases over time, hence the importance of sequential booster doses [17].

Therefore, it is essential to monitor the longitudinal kinetics of antibodies against SARS-CoV-2 in vaccinated individuals, aiming to evaluate the immunization scheme established by regulatory institutions. It is necessary to understand the length of time the organism takes to develop neutralizing antibodies with the stipulated vaccine doses and the duration of the acquired immunity. The information obtained will be crucial in guiding health authorities on the necessity of administering future booster doses and the best time for this.

To contribute to understanding the specific antibody response to SARS-CoV-2 in the population, our study aimed to monitor the production and maintenance of neutralizing antibodies and anti-SARS-CoV-2 spike RBD protein in individuals after primary vaccination with BNT162b2 or ChAdOx1 and two doses of homologous or heterologous boosters. Furthermore, we tracked the neutralizing antibody response against the variants of concern (VOCs), as follows: Gamma and Delta.

## 2. Materials and Methods

### 2.1. Ethical Aspects, Study Population, and Kinetics

This prospective cohort study was conducted between March 2021 and March 2023 at the Evandro Chagas Institute (IEC), Ananindeua, Pará state, Brazil. This study was approved by the Institutional Research Ethics Committee (CAAE 43109021.7.0000.0019), and all participants signed the informed consent form. The cohort comprised 40 individuals who were starting the vaccination scheme against COVID-19, residents of the metropolitan region of Belém, State of Pará, Brazil, who were recruited through institutional information about voluntary participation in the project. We divided the studied population into two groups according to the primary vaccination scheme (i.e., 1st and 2nd doses). The characteristics of the participants are detailed in Table 1.

**Figure 1:**
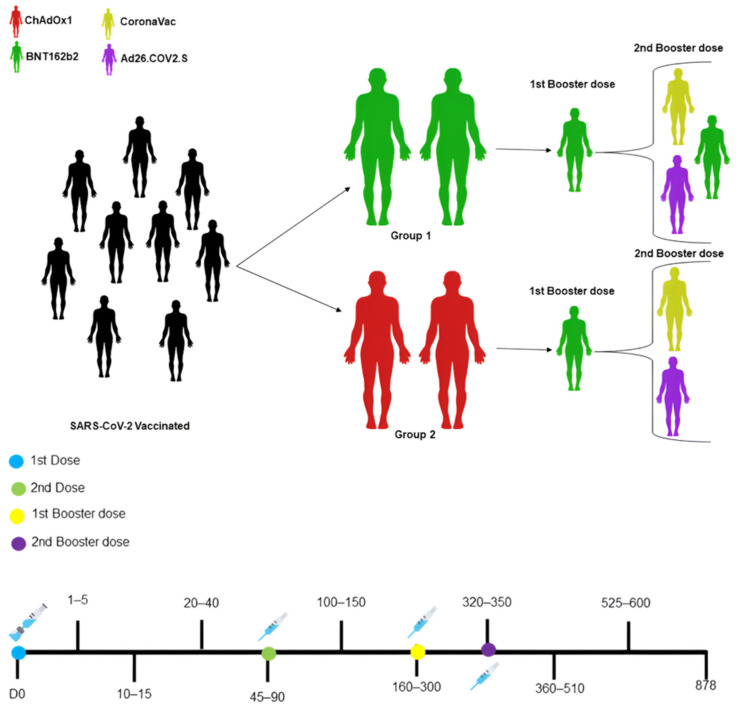
Schematic representation of the groups and vaccines administered.

In group 1 (n = 22), all participants received the BNT162b2 vaccine in their primary vaccination scheme. In the secondary vaccination scheme, everyone received the BNT162b2 vaccine in the first booster (3rd dose), while twelve participants were vaccinated with CoronaVac, four with Ad26.COV2.S, and four with BNT162b2 in the second booster (4th dose) (Figure 1).

In group 2 (n = 18), in the primary vaccination scheme, all participants received ChAdOx1 (Figure 1), with a first booster dose of BNT162b2. In the second booster, eleven participants received CoronaVac, two received Ad26.COV2.S, and four individuals had not yet received this 4th dose during the evaluation. Blood collections in both groups were carried out immediately before immunization, day 0 (baseline), and serially postimmunization, between days 1 and 5, 10 and 15, 20 and 40, 45 and 90, 100 and 150, 160 and 300, 320 and 350, 360 and 510, 525 and 600, and on 878 (Figure 1). All samples were centrifuged at 800 rpm for 10 min to obtain serum and then stored at −80 °C for later analysis.

### 2.2. Indirect Enzyme Immunoassay (ELISA) Specific for SARS-CoV-2 RBD

The receptor-binding domain (RBD) of the spike protein from the ancestral strain of SARS-CoV-2 (GenBank accession number: MN908947, residues 319–524) was expressed and purified as described by Rawle and colleagues (2021) [18].

The samples were tested for the detection of total anti-RBD SARS-CoV-2 antibodies by indirect ELISA, as described by Oliveira and colleagues (2022) [17] Briefly, 96-well plates (Thermo Fisher Scientific, Waltham, MA, USA) were coated with 50 µL/well of 2 µg/mL RBD in 0.1 M pH 7.4 buffered saline (PBS) and incubated overnight at 4 °C. The next day, after removal of the coating by three washes with distilled water, we added 150 µL/well of blocking buffer solution (KPL Milk diluent/Blocking solution concentrate (SeraCare, Milford, MA, USA), diluted in PBS containing 0.5% Tween 20) and stored for one hour at room temperature.

After the blocking step, the plates were washed three times by submersion in distilled water, and we added 50 µL/well of diluted serum samples (1:100 in blocking buffer) and controls (positive and negative human sera) to the plates and incubated for one hour at 37 °C. Following this, the plates were washed with distilled water three times before the addition of 50 µL/well of goat anti-human kappa secondary antibody (Sigma Aldrich, Saint Louis, MO, USA) diluted to 1:2000. The plates were incubated for one hour at 37 °C. Next, the unbound secondary antibody was removed by three washes with distilled water, and 50 µL/well of HRP-conjugated polyclonal anti-goat antibody conjugated to peroxidase (HRP) (DAKO, Glostrup, Denmark) diluted to 1:1000 was added and incubated for one hour at 37 °C.

Then, three washes were performed to remove all of the unbound polyclonal antibodies. Then, 50 µL/well of the substrate Tetramethylbenzidine (TMB) (SeraCare, Milford, MA, USA) was added and stored in a dark chamber at room temperature (25 °C) for 5 min. We stopped the reaction by adding 30 µL/well of stop solution (1 M H_2_SO_4_). The optical density (OD) at 450 nm was then read using the LMR-96 microplate reader (Loccus Biotecnologia, Cotia, Brazil). The OD of the blank was subtracted from each sample, and the cutoff was determined using the ROC curve. Samples with antibodies with an OD ≥ 0.200 were considered positive.

### 2.3. Plaque Reduction Neutralization Test 50% (PRNT50)

Eight participants from the most homogeneous group (group 1) were randomly chosen to determine the neutralizing antibody titers based on PRNT50 against the Gamma (P1) and Delta variants, collected before (baseline, D0) and after vaccination (1–878 days). The PRNT50 determination was performed as described by Roehrig and colleagues (2008) [19] with adaptations. Briefly, the samples from CoronaVac-vaccinated individuals and previously selected controls (positive and negative human sera) were heat-inactivated (56 °C for one hour), and two-fold serial dilutions (1:20–1:1280) were performed in maintenance medium 199 (M199). Next, the diluted sera were incubated with the SARS-CoV-2 variants at 37 °C and 5% CO_2_ for one hour.

We used equal amounts of virus suspension containing 100 PFU for the serum–virus complexes. Then, the immunocomplexes were added to 24-well plates containing a confluent monolayer of Vero CCL-81 cells (ATCC, Manassas, VA, USA) and incubated for one hour at 37 °C and 5% CO_2_. Subsequently, we added 1.5 mL of the overlay medium (medium 199 containing 1.5% carboxymethylcellulose and 5% fetal bovine serum (FBS)) to each well and incubated for four days at 37 °C in an atmosphere containing 5% CO_2_. The plates were fixed using 10% formaldehyde for four hours and then stained with 1% Crystal Violet for two hours.

Plaques were counted under an optical microscope (Zeiss, Oberkochen, Germany). After counting the plaques, the neutralizing antibody titers were determined based on the PRNT50, considering the positive sera that reduced the number of plaques by 50% compared to the average of the virus control wells.

### 2.4. Statistical Analysis

All statistical analyses and graphical representations were performed using GraphPad Prism software (version 9.0, San Diego, CA, USA). The Mann–Whitney U test was used to evaluate the statistical difference between group 1 and group 2 in relation to the fourth dose of the CoronaVac vaccine, comparing the group of immunized individuals who had a previous natural infection versus individuals without a previous natural infection. These groups were stratified by days after vaccination. The statistical difference was evaluated by multiple comparisons of the group mean using two-way ANOVA tests, followed by Tukey’s post hoc tests. For all analyses, statistical significance was assumed when *p* < 0.05. The mean ± standard deviation (SD) was calculated to compare the obtained optical density (OD) values and/or viral neutralization titers. Linear regression and Spearman’s correlation were used to evaluate the strength and power of correction between the OD values obtained and the neutralization titers.

## 3. Results

### 3.1. Assessment of the Anti-SARS-CoV-2 Antibody Response: Primary and Booster Doses

#### 3.1.1. Assessment of Total Anti-RBD Antibody Levels in Groups 1 (BNT162b2) and 2 (ChAdOx1)

**Figure 2:**
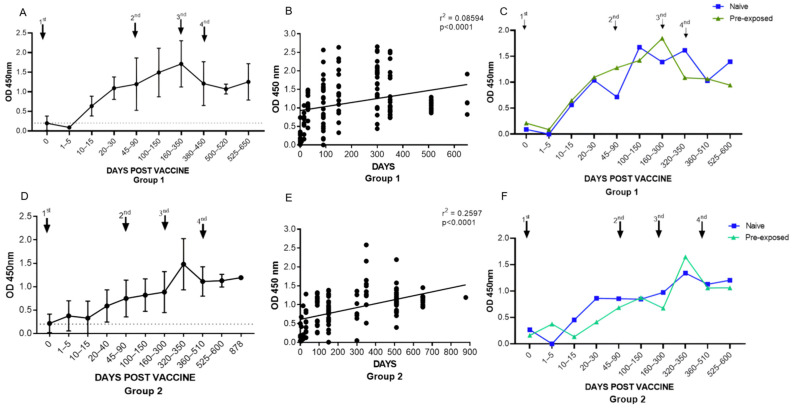
Monitoring of anti-RBD SARS-CoV-2 specific antibodies by indirect enzyme immunoassay (ELISA) in Group 1 and Group 2: (**A**) curve of specific antibodies for RBD (Group 1), expressed as the mean ± standard deviation; (**B**) linear regression analysis of anti-RBD antibodies along the experimental kinetics (Group 1); (**C**) curve of antibodies specific to RBD of those vaccinated (Group 1) who were infected naturally or not by SARS-CoV-2; (**D**) curve of antibodies specific to RBD (Group 2), expressed as the mean ± standard deviation; (**E**) linear regression analysis of anti-RBD antibodies over time (Group 2); (**F**) curve of antibodies specific to RBD of individuals in Group 2 who were infected naturally or not with SARS-CoV-2.

While monitoring the total anti-RBD antibody response throughout the experimental kinetics in group 1, we observed a progressive growth, with the average OD levels varying between 0.196 and 1.712. A linear regression analysis (Figure 2B) confirmed this progression, with a positive relationship in the anti-RBD antibody titers (r2 = 0.08594, *p* < 0.0001). It was possible to observe average antibody levels from 10 to 15 days after the 1st dose; however, seroconversion of the entire group occurred around days 20–30, with an average OD of 1.092. The titers continued to increase with a maximum average detected after the first booster (3rd dose), between days 160 and 300, followed by a decline in the titers between days 320 and 350. After the second booster (4th dose, with Ad26.COV2.S, BNT162b2, or CoronaVac), the antibody levels remained more stable until the end of follow-up (Figure 2A). When analyzing the profile of individuals in group 1 who were naturally infected by SARS-CoV-2 and those not naturally infected, we observed that there was no statistical difference between them, although higher average titers were observed almost throughout the monitoring period in individuals previously infected with SARS-CoV-2 (Figure 2C).

In group 2, we also observed a gradual increase in anti-RBD antibodies with a positive relationship in the levels of anti-RBD antibody titers confirmed by linear regression analysis (r2 = 0.2597, *p* < 0.0001) (Figure 2E). The mean OD titers ranged from 0.216 to 1.480, with mean levels detectable between 5 and 15 days after the 1st dose. The total seroconversion of the group occurred after the 2nd dose (45–90 days) with an average OD of 0.748, with a maximum mean titer observed (OD 1.480) after the first booster (3rd dose) with BNT162b2, followed by a subtle decline that stabilizes right after the second booster (4th dose, with Ad26.COV2.S or CoronaVac). Regarding the profile of the individuals in group 2 who had a natural infection or not by SARS-CoV-2, we did not observe a statistical difference between them throughout the follow-up (Figure 2F).

#### 3.1.2. Assessment of Total Anti-RBD Antibody Levels after the Second Booster with the Different Vaccines Administered

**Figure 3:**
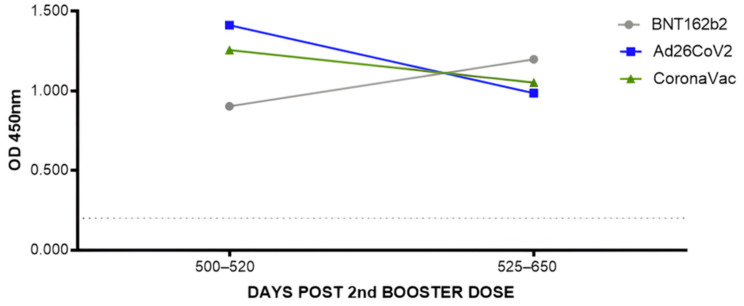
Monitoring anti-RBD antibodies after administration of the second booster in group 1.

To analyze anti-RBD antibody levels after the second booster, we stratified group 1 and analyzed the profile separately according to the type of vaccine administered. We observed a subtle increase in antibody levels in individuals who received the second homologous booster with the BNT162b2 vaccine. In contrast, individuals who received the second heterologous booster with the Ad26.COV.2 and CoronaVac vaccines showed a slight decrease in anti-RBD antibody levels. However, all three vaccines administered in the second booster reached similar average OD levels, which were not significant among them (Figure 3). This analysis was not carried out with group 2, as only two participants received the second booster with Ad26COV2. All other participants received CoronaVac.

#### 3.1.3. Assessment of Total Anti-RBD Antibody Levels between Group 1 (BNT162b2) and Group 2 (ChAdOx1)

**Figure 4:**
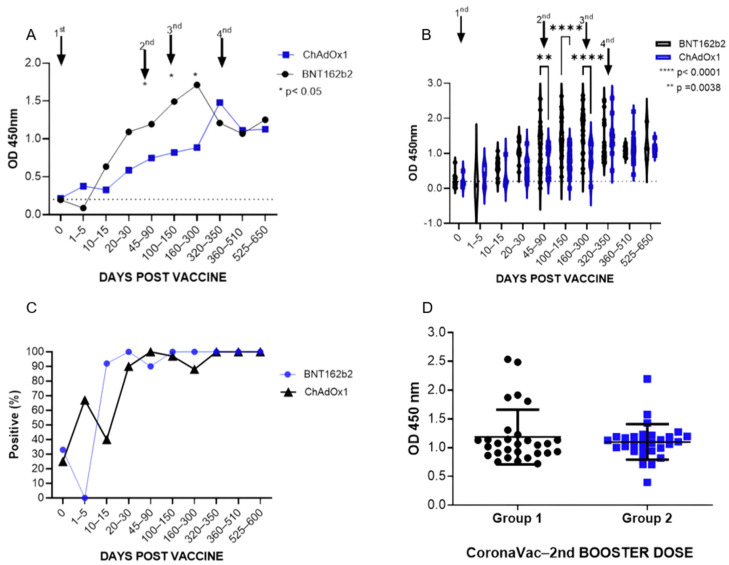
Total anti-RBD antibody levels were assessed between groups 1 (BNT162b2) and 2 (ChAdOx1), expressed as the mean OD ± standard deviation: (**A**) comparative analysis between group 1 and group 2 of the mean OD values in the study with a complete vaccination scheme (primary vaccination scheme + boosters), * *p* < 0.05; (**B**) distribution of the OD values per day as a dot plot, **** *p* < 0.0001 and ** *p* = 0.0038; (**C**) percentage of seroconversion in individuals from group 1 and group 2; (**D**) comparison of the OD distribution of groups 1 and 2 with the second booster dose with the CoronaVac vaccine.

**Figure 5:**
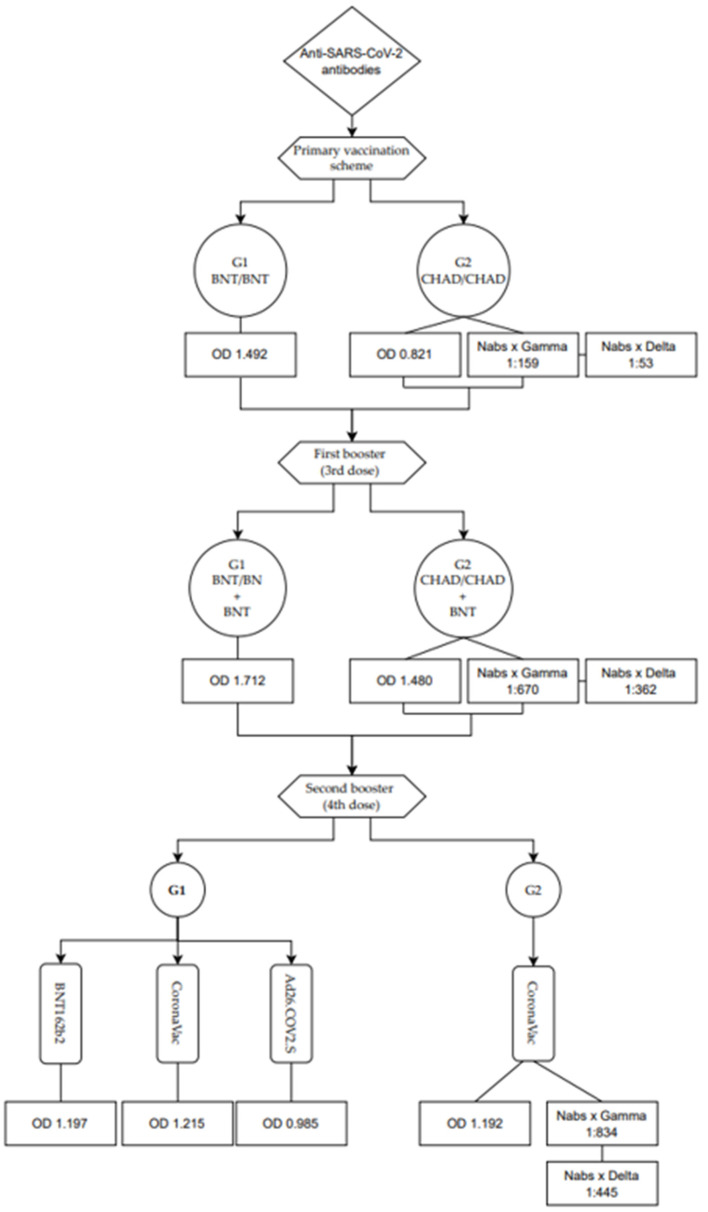
Levels of anti-SARS-CoV-2 antibodies from the primary vaccination scheme to the second booster, expressed as the mean OD and as the average by PRNT50 with the Gamma and Delta variants. BNT, BNT162b2; CHAD, ChAdOx1; Nabs, neutralizing antibodies.

To better understand the dynamics of anti-RBD antibodies, we decided to compare the results obtained with ELISA from the two established groups based on the primary vaccination scheme, group 1 (BNT162b2) and group 2 (ChAdOx1). Our analysis demonstrated higher levels in group 1 for almost the entire period of the kinetics but with a significant difference only after the second dose of the primary regimen, at 90 days (*p* = 0.0038), and in the first booster (3rd dose), at 150 and 300 days (*p* < 0.001) of follow-up (Figure 4B). We also observed that right after the second booster (4th dose), at around 510 and 650 days, group 1 and group 2 showed similar titers (Figure 4A and Figure 5). Concerning positivity, it was also possible to observe a difference between these groups, where group 2 achieved 100% seroconversion only after the influence of the 2nd dose (90 days), while group 1, at between 10 and 15 days, had a positivity rate of around 92%, reaching 100% between 20 and 30 days after the 1st dose (Figure 4C).

When comparing the two groups after the second booster (4th dose), stratifying only the individuals who received the CoronaVac vaccine, the statistical analysis showed no significant difference between them (Figure 4D and Figure 5).

#### 3.1.4. Levels of Neutralizing Antibodies in Individuals Who Received the ChadOx1 Vaccine in the Primary Vaccination Scheme, First Booster with BNT162b2, and Second Booster with CoronaVac (Group 2)

**Figure 6:**
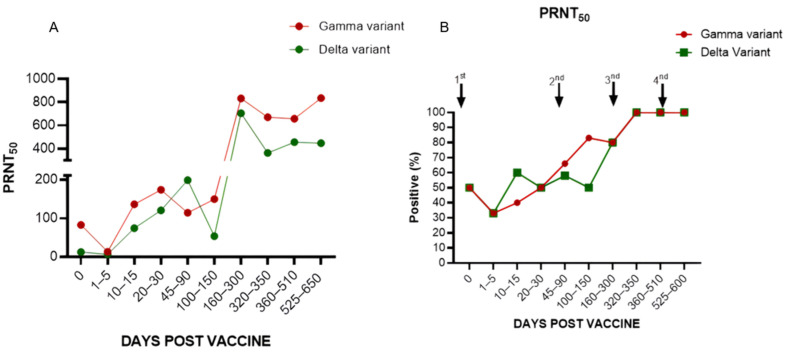
(**A**) Levels of neutralizing antibodies in individuals from group 2 against the Gamma (P1) and Delta variants (values expressed as the average by the PRNT50); (**B**) percentage of seroconversion in individuals analyzed by PRNT.

We analyzed the concentrations of anti-SARS-CoV-2 neutralizing antibodies against two variants, Gamma and Delta, in participants (n = 8) from group 2. It was possible to observe, compared to the Gamma variant, more expressive titers between 20 and 30 days after the 1st dose, with an average titer of 1:173 and subtle growth after the 2nd dose; a relevant increase was observed after the first booster, with an average of titer 1:834 (Figure 5 and Figure 6A), as well as a 100% seropositivity rate that was maintained until the end of follow-up (Figure 6B).

The same anti-SARS-CoV-2 neutralizing antibody response profile was observed regarding the Delta variant but with lower titers. Between 20 and 30 days after the 1st dose, the mean titer was 1:120, with an increase observed after the first booster and a mean titer of 704, followed by a decrease in mean titers even after the second booster, 1:445, which was maintained until the end of follow-up (Figure 5 and Figure 6A).

## 4. Discussion

As vaccination campaigns gained speed, knowing what level of neutralizing antibodies is associated with the vaccine-induced immunological persistence and the need for boosters are questions raised that make evident the need for longitudinal studies in different populations, as well as with various vaccine platforms between homologous and heterologous boosters.

Given the rapid evolution of SARS-CoV-2 and the emergence of new variants of concern, several studies have demonstrated the need for additional booster doses regardless of the primary vaccination scheme used [20,21]. In our study, we observed a positive progression in the mean levels of anti-RBD antibodies, confirmed by linear regression analysis in both groups. However, we observed higher mean titers in group 1 since the primary regimen with BNT162b2 significantly differed after the 2nd and 3rd doses (1st booster). Other evidence that suggests a greater and faster immune system stimulation by this vaccine platform is the fact that 100% seropositivity was achieved 20–30 days after the 1st dose. At the same time, in group 2, this positivity was only observed after the 2nd dose of ChAdOx1. Although it is not possible to state that the technology used in the production of vaccines is the main reason for the variation in effectiveness, numerous comparative studies have demonstrated that human vaccination based on SARS-CoV-2 mRNA induces a persistent response from B cells of the germinal center, capable of generating a robust humoral immunity response [22,23,24,25].

When analyzing the profiles of individuals who had or had not been infected naturally with SARS-CoV-2 before primary vaccination, studies with different vaccine platforms report that pre-exposure to infection increases the initial antibody response after immunization compared to naïve individuals [17,26,27,28]. However, in our study, no statistical difference was observed between naïve and previously infected individuals in both groups analyzed; it is worth highlighting that most pre-exposed individuals, group 1 and group 2, were infected with SARS-CoV-2 between 11 and 12 months before the vaccination, showing no more seropositivity in the anti-RBD ELISA. This finding was presented as a determinant for the similarity of anti-RBD antibody response, as well as neutralizing antibodies between the pre-exposed and naïve population in a study with CoronaVac [17], suggesting that the production of higher antibody titers observed in pre-exposed individuals was generated exclusively by those who had detectable anti-RBD antibodies in the ELISA before vaccination (i.e., baseline).

Several observational studies have already reported on short-term vaccine immunogenicity after primary regimens or 1st booster, demonstrating that heterologous regimens consistently achieve higher immunogenicity when inactivated vaccines are administered before or after vectorized or mRNA vaccines [24,29,30], as well as when vectorized vaccines are administered before or after mRNA vaccines [31,32,33,34]. In our study, both groups were vaccinated with BNT162b2 in the 1st booster (3rd dose), showing a more significant increase in anti-RBD antibodies in group 2, characterized by a heterologous booster.

Most studies with BNT162b2, comparing homologous and heterologous regimens, have not demonstrated clear evidence of higher immunogenicity when this vaccine is administered before or after vectorized vaccines, indicating in these cases an approximate equivalence of heterologous and homologous regimens [31]. It is important to mention that most of the studies describe immunogenicity in a shorter postboost period, which probably still reflects the high levels of antibodies originating from the primary regimen with BNT162b2, which are significantly higher than with ChAdOx1 [24,35]. Nevertheless, our results show decreased anti-RBD and neutralizing antibodies after homologous reinforcement in group 1. This is probably due to the analyses being carried out 6–7 months after the booster, which is enough time for these antibodies to decline. However, it is worth highlighting that despite this decline in antibodies obtained with the homologous booster (group 1), the levels observed are considered high and do not characterize a decrease in vaccine effectiveness. In agreement with our heterologous regimen results, Liu and colleagues (2023) [33] observed a higher and more durable humoral response profile in heterologous boosters between BNT162b2 and ChAdOx1 up to 8 months after the 3rd dose.

After the 2nd booster, groups 1 and 2 showed more stable and similar average levels of anti-RBD antibodies until the end of the kinetics, five months after this 4th dose. Several studies have observed this similarity in antibody levels at a given time after the 2nd dose of the primary regimen or additional doses between homologous or heterologous boosters with different vaccine platforms and still between profiles of individuals with or without a history of previous infection [36,37], which may cause this booster dose to raise the titers only up to a certain level independent of the initial titers at the starting point [35]. Their results correlate with our observations after the 3rd dose of little or no anti-RBD antibody response in group 1, which already maintained high concentrations before this booster and would probably have overtaken this maximum “level”, as well as in group 2, as the increase in antibody concentrations was possible as they would not have yet reached this expected “level”. This event may be related to a possible stabilization of the stimulus generated by the vaccine doses in the production of antibodies. It would explain why the average final antibody titers in the different study groups were similar.

When we stratified group 1 according to the type of vaccine administered in the 2nd booster, analyzing the last two time points corresponding to 2–3 months and five months after the 4th dose, it was also possible to observe variations in the average anti-RBD antibody titers among the three vaccine platforms to reach a certain “level” of OD that was similar without showing a statistical difference between them.

A relevant fact to be presented is that in both groups, even after five months of the 2nd booster, the average levels of anti-RBD antibodies remained stabilized in concentrations considered high, even with CoronaVac, which has already demonstrated a significant decline in anti-RBD and neutralizing antibodies in the same period after the booster in the primary scheme (2nd dose) [17,38,39,40], including not being recommended as a 1st booster after the primary scheme (3rd dose) [41]. This change in profile may be related to the maturation of memory B cells acquired during the pandemic, which involved some factors including subsequent doses of immunizing agents. The increase in the affinity of the immunoglobulin to its specific antigen is possible through affinity maturation which occurs predominantly after the second and third exposures to the antigen [42].

In individuals who received the ChadOx1 vaccine in the primary vaccination scheme, a first booster with BNT162b2 and a second booster with CoronaVac (group 2), when we analyzed the concentrations of neutralizing antibodies against the two variants, Gamma and Delta, our results indicate the maintenance of high concentrations until the end of follow-up. For both variants, we observed similar anti-SARS-CoV-2 neutralizing antibody responses, although the Delta variant demonstrated titers twice as low even with 100% seropositivity since the 3rd dose.

These superior responses by neutralizing antibodies, which were longer lasting, may also be related to the epidemiological scenario during the pandemic, whereby natural exposure to several circulating SARS-CoV-2 variants increases antibody levels, including heterologous antibodies that will contribute to immunity. It is worth mentioning that in Para state, Brazil, there was a long circulation period of the Gamma variant and its subunits. In January 2022, when this variant still predominated [43], almost one-third of the participants in group 2 became naturally infected shortly before receiving the 3rd dose. This fact may have favored the significant increase in neutralizing antibodies after this booster, which is related to the heterogeneity of the SARS-CoV-2 neutralization activities after infection or vaccination [44].

One of the limitations of our study was the small sample size, a difficulty found in independent longitudinal studies. Furthermore, we were unable to evaluate postvaccination neutralizing antibodies against currently circulating VOCs such as Omicron and its subvariants [45]. These data could confirm or not the reduction in efficacy in the face of this VOC of neutralizing antibodies generated by the 2nd dose of heterologous vaccine booster after an extended period. It is known that the most significant characteristic of Omicron was the immune escape from neutralizing antibodies generated by the vaccine (evolutionary process) [46]; however, it was observed by Wietschel et al. (2024) [47] that the 1st homologous booster with BNT was capable of inducing neutralizing antibodies against the Omicron BA.2 variant. Another point to be investigated is the effectiveness of successive vaccine doses in different groups, as well as the long-term monitoring of neutralizing antibodies in individuals who received the bivalent vaccine.

## 5. Conclusions

In conclusion, it is possible to say that the circulating levels of RBD-specific antibodies in individuals after primary vaccination with BNT162b2 or ChAdOx1 and two doses of homologous boosters, BNT162b2, or heterologous, CoronaVac and Ad26.COV2.S, are durable, with significant average titers even five months after the 2nd booster. The same profile was observed with neutralizing antibodies without a substantial decrease facing the VOC Delta, suggesting an increase in the affinity of the immunoglobulin to its specific antigen.

## Figures and Tables

**Table 1:** General characteristics of the studied population.

Variables	Group 1	Group 2
Gender		
Female	12 (54.5%)	12 (66.6%)
Male	10 (45.4%)	6 (33.3%)
Age Group		
Means (±)	39.8 (±7.5)	39.6 (±12.4)
Median	38.5	39.0
Natural Infection		
Symptomatic	17 (77.2%)	9 (50%)
Asymptomatic	5 (22.7%)	9 (50%)
Comorbidities		
Psoriasis	-	1 (5.5%)
Arterial Hypertension	-	1 (5.5%)
Obesity	-	1 (5.5%)
Type 2 diabetes	1 (4.5%)	-

## Data Availability

The data presented in this study are available in this article.

## References

[1] Lu R., Zhao X., Li J., Niu P., Yang B., Wu H., Wang W., Song H., Huang B., Zhu N. (2020). Genomic Characterisation and Epidemiology of 2019 Novel Coronavirus: Implications for Virus Origins and Receptor Binding. Lancet.

[2] Queiroz M.A.F., Santiago A.M., de Brito W.B., Pereira K.A.S., de Brito W.B., da Silva Torres M.K., da Costa Lopes J., dos Santos E.F., da Costa F.P., de Sarges K.M.L. (2023). Polymorphisms in the MBL2 Gene Are Associated with the Plasma Levels of MBL and the Cytokines IL-6 and TNF-α in Severe COVID-19. Front. Immunol..

[3] Rodrigues F.B.B., da Silva R., Santos E.F.d., de Brito M.T.F.M., da Silva A.L.S., de Meira Leite M., Póvoa da Costa F., de Nazaré do Socorro de Almeida Viana M., de Sarges K.M.L., Cantanhede M.H.D. (2023). Association of Polymorphisms of IL-6 Pathway Genes (IL6, IL6R and IL6ST) with COVID-19 Severity in an Amazonian Population. Viruses.

[4] Malik Y.A. (2020). Properties of Coronavirus and SARS-CoV-2. Malays. J. Pathol..

[5] Chilamakuri R., Agarwal S. (2021). COVID-19: Characteristics and Therapeutics. Cells.

[6] Raskin S. (2021). Genetics of COVID-19. J. Pediatr..

[7] Beavis K.G., Matushek S.M., Abeleda A.P.F., Bethel C., Hunt C., Gillen S., Moran A., Tesic V. (2020). Evaluation of the EUROIMMUN Anti-SARS-CoV-2 ELISA Assay for Detection of IgA and IgG Antibodies. J. Clin. Virol..

[8] Tré-Hardy M., Wilmet A., Beukinga I., Favresse J., Dogné J., Douxfils J., Blairon L. (2021). Analytical and Clinical Validation of an ELISA for Specific SARS-CoV-2 IgG, IgA, and IgM Antibodies. J. Med. Virol..

[9] Padhi A.K., Tripathi T. (2020). Can SARS-CoV-2 Accumulate Mutations in the S-Protein to Increase Pathogenicity?. ACS Pharmacol. Transl. Sci..

[10] Noureddine F.Y., Chakkour M., El Roz A., Reda J., Al Sahily R., Assi A., Joma M., Salami H., Hashem S.J., Harb B. (2021). The Emergence of SARS-CoV-2 Variant(s) and Its Impact on the Prevalence of COVID-19 Cases in the Nabatieh Region, Lebanon. Med. Sci..

[11] Martínez-Anaya C., Ramos-Cervantes P., Vidaltamayo R. (2020). Coronavirus, Diagnosis and Epidemiological Strategies against COVID-19 in Mexico. Educ. Quim..

[12] Chmielewska B., Barratt I., Townsend R., Kalafat E., van der Meulen J., Gurol-Urganci I., O’Brien P., Morris E., Draycott T., Thangaratinam S. (2021). Effects of the COVID-19 Pandemic on Maternal and Perinatal Outcomes: A Systematic Review and Meta-Analysis. Lancet Glob. Health.

[13] Ita K. (2021). Coronavirus Disease (COVID-19): Current Status and Prospects for Drug and Vaccine Development. Arch. Med. Res..

[14] Munro A.P.S., Janani L., Cornelius V., Aley P.K., Babbage G., Baxter D., Bula M., Cathie K., Chatterjee K., Dodd K. (2021). Safety and Immunogenicity of Seven COVID-19 Vaccines as a Third Dose (Booster) Following Two Doses of ChAdOx1 NCov-19 or BNT162b2 in the UK (COV-BOOST): A Blinded, Multicentre, Randomised, Controlled, Phase 2 Trial. Lancet.

[15] Mahdi P.D.B.M., Almukhtar D.M. (2023). Role Of Vaccines Against COVID-19 Pandemic. SLAS Discov..

[16] Boulware D.R., Lindsell C.J., Stewart T.G., Hernandez A.F., Collins S., McCarthy M.W., Jayaweera D., Gentile N., Castro M., Sulkowski M. (2023). Inhaled Fluticasone Furoate for Outpatient Treatment of COVID-19. N. Engl. J. Med..

[17] de Oliveira C.F., Neto W.F.F., da Silva C.P., Ribeiro A.C.S., Martins L.C., de Sousa A.W., Freitas M.N.O., Chiang J.O., Silva F.A., Dos Santos E.B. (2022). Absence of Anti-RBD Antibodies in SARS-CoV-2 Infected or Naive Individuals Prior to Vaccination with CoronaVac Leads to Short Protection of Only Four Months Duration. Vaccines.

[18] Rawle D.J., Le T.T., Dumenil T., Yan K., Tang B., Nguyen W., Watterson D., Modhiran N., Hobson-Peters J., Bishop C. (2021). ACE2-Lentiviral Transduction Enables Mouse SARS-CoV-2 Infection and Mapping of Receptor Interactions. PLoS Pathog..

[19] Roehrig J.T., Hombach J., Barrett A.D.T. (2008). Guidelines for Plaque-Reduction Neutralization Testing of Human Antibodies to Dengue Viruses. Viral Immunol..

[20] McLean G., Kamil J., Lee B., Moore P., Schulz T.F., Muik A., Sahin U., Türeci Ö., Pather S. (2022). The Impact of Evolving SARS-CoV-2 Mutations and Variants on COVID-19 Vaccines. mBio.

[21] Firouzabadi N., Ghasemiyeh P., Moradishooli F., Mohammadi-Samani S. (2023). Update on the Effectiveness of COVID-19 Vaccines on Different Variants of SARS-CoV-2. Int. Immunopharmacol..

[22] Lederer K., Castaño D., Gómez Atria D., Oguin T.H., Wang S., Manzoni T.B., Muramatsu H., Hogan M.J., Amanat F., Cherubin P. (2020). SARS-CoV-2 MRNA Vaccines Foster Potent Antigen-Specific Germinal Center Responses Associated with Neutralizing Antibody Generation. Immunity.

[23] Turner J.S., O’Halloran J.A., Kalaidina E., Kim W., Schmitz A.J., Zhou J.Q., Lei T., Thapa M., Chen R.E., Case J.B. (2021). SARS-CoV-2 MRNA Vaccines Induce Persistent Human Germinal Centre Responses. Nature.

[24] Costa Clemens S.A., Marchevsky N., Kelly S., Felle S., Eldawi A., Rajasingam R., Mahmud R., Lambe T., Voysey M., Gonzalez I. (2023). Immunogenicity, Safety and Reactogenicity of Heterologous (Third Dose) Booster Vaccination with a Full or Fractional Dose of Two Different COVID-19 Vaccines: A Phase 4, Single-Blind, Randomized Controlled Trial in Adults. Hum. Vaccines Immunother..

[25] Braga A., Tura B., Alberto C., Magliano S., Senna K., Macedo L.S., Santos M., Padilla M.P., Fernandes R. (2021). Vacina Da Fiocruz [ChAdOx-1 (Vacina COVID-19 Recombinante)] e Da Pfizer/Wyeth [BNT162b2 (Vacina COVID-19)] Para Prevenção Da COVID-19.

[26] Long Q.X., Tang X.J., Shi Q.L., Li Q., Deng H.J., Yuan J., Hu J.L., Xu W., Zhang Y., Lv F.J. (2020). Clinical and Immunological Assessment of Asymptomatic SARS-CoV-2 Infections. Nat. Med..

[27] Goel R.R., Painter M.M., Apostolidis S.A., Mathew D., Meng W., Rosenfeld A.M., Lundgreen K.A., Reynaldi A., Khoury D.S., Pattekar A. (2021). MRNA Vaccines Induce Durable Immune Memory to SARS-CoV-2 and Variants of Concern. Science.

[28] Ebinger J.E., Fert-Bober J., Printsev I., Wu M., Sun N., Prostko J.C., Frias E.C., Stewart J.L., Van Eyk J.E., Braun J.G. (2021). Antibody Responses to the BNT162b2 MRNA Vaccine in Individuals Previously Infected with SARS-CoV-2. Nat. Med..

[29] Costa Clemens S.A., Weckx L., Clemens R., Almeida Mendes A.V., Ramos Souza A., Silveira M.B.V., da Guarda S.N.F., de Nobrega M.M., de Moraes Pinto M.I., Gonzalez I.G.S. (2022). Heterologous versus Homologous COVID-19 Booster Vaccination in Previous Recipients of Two Doses of CoronaVac COVID-19 Vaccine in Brazil (RHH-001): A Phase 4, Non-Inferiority, Single Blind, Randomised Study. Lancet.

[30] Nanthapisal S., Puthanakit T., Jaru-Ampornpan P., Nantanee R., Sodsai P., Himananto O., Sophonphan J., Suchartlikitwong P., Hiransuthikul N., Angkasekwinai P. (2022). A Randomized Clinical Trial of a Booster Dose with Low versus Standard Dose of AZD1222 in Adult after 2 Doses of Inactivated Vaccines. Vaccine.

[31] Lv J., Wu H., Xu J., Liu J. (2022). Immunogenicity and Safety of Heterologous versus Homologous Prime-Boost Schedules with an Adenoviral Vectored and MRNA COVID-19 Vaccine: A Systematic Review. Infect. Dis. Poverty.

[32] Deng J., Ma Y., Liu Q., Du M., Liu M., Liu J. (2022). Comparison of the Effectiveness and Safety of Heterologous Booster Doses with Homologous Booster Doses for SARS-CoV-2 Vaccines: A Systematic Review and Meta-Analysis. Int. J. Environ. Res. Public Health.

[33] Liu X., Munro A.P.S., Wright A., Feng S., Janani L., Aley P.K., Babbage G., Baker J., Baxter D., Bawa T. (2023). Persistence of Immune Responses after Heterologous and Homologous Third COVID-19 Vaccine Dose Schedules in the UK: Eight-Month Analyses of the COV-BOOST Trial. J. Infect..

[34] Okuyama R. (2023). MRNA and Adenoviral Vector Vaccine Platforms Utilized in COVID-19 Vaccines: Technologies, Ecosystem, and Future Directions. Vaccines.

[35] Fadlyana E., Setiabudi D., Kartasasmita C.B., Putri N.D., Rezeki Hadinegoro S., Mulholland K., Sofiatin Y., Suryadinata H., Hartantri Y., Sukandar H. (2023). Immunogenicity and Safety in Healthy Adults of Full Dose versus Half Doses of COVID-19 Vaccine (ChAdOx1-S or BNT162b2) or Full-Dose CoronaVac Administered as a Booster Dose after Priming with CoronaVac: A Randomised, Observer-Masked, Controlled Trial in Indonesia. Lancet Infect. Dis..

[36] Taniguchi Y., Suemori K., Tanaka K., Okamoto A., Murakami A., Miyamoto H., Takasuka Y., Yamashita M., Takenaka K. (2023). Long-Term Transition of Antibody Titers in Healthcare Workers Following the First to Fourth Doses of MRNA COVID-19 Vaccine: Comparison of Two Automated SARS-CoV-2 Immunoassays. J. Infect. Chemother..

[37] Matsuura T., Fukushima W., Nakagama Y., Kido Y., Kase T., Kondo K., Kaku N., Matsumoto K., Suita A., Mukai E. (2024). Factors Impacting Antibody Kinetics, Including Fever and Vaccination Intervals, in SARS-CoV-2-Naïve Adults Receiving the First Four MRNA COVID-19 Vaccine Doses. Sci. Rep..

[38] Xu Q.Y., Xue J.H., Xiao Y., Jia Z.J., Wu M.J., Liu Y.Y., Li W.L., Liang X.M., Yang T.C. (2021). Response and Duration of Serum Anti-SARS-CoV-2 Antibodies after Inactivated Vaccination within 160 Days. Front. Immunol..

[39] Harapan H., Ar Royan H., Tyas I.I., Nadira A., Abdi I.F., Anwar S., Husnah M., Ichsan I., Pranata A., Mudatsir M. (2022). Waning Anti-SARS-CoV-2 Neutralizing Antibody in CoronaVac-Vaccinated Individuals in Indonesia. F1000Res.

[40] Volpe G.J., Vessoni S.C.S., Soares L.B., Leite dos Santos Almeida M.A.A., Braga P.E., de Moraes G.R., Ferreira N.N., Garibaldi P.M.M., Kashima S., Fonseca B.A.L. (2023). Antibody Response Dynamics to CoronaVac Vaccine and Booster Immunization in Adults and the Elderly: A Long-Term, Longitudinal Prospective Study. IJID Reg..

[41] ANVISA (2021). VOTO No. 214/2021/SEI/DIRE2/ANVISA: Avaliação Da Anvisa Sobre Doses de Reforço Para as Vacinas Contra a COVID-19.

[42] Abbas A.K., Lichtman A.H., Pillai S. (2019). Ativação da Célula B e Produção de Anticorpos. Imunologia Celular e Molecular.

[43] Pinho C.T., Vidal A.F., Negri Rocha T.C., Oliveira R.R.M., da Costa Barros M.C., Closset L., Azevedo-Pinheiro J., Braga-da-Silva C., Silva C.S., Magalhães L.L. (2023). Transmission Dynamics of SARS-CoV-2 Variants in the Brazilian State of Pará. Front. Public Health.

[44] Graninger M., Camp J.V., Aberle S.W., Traugott M.T., Hoepler W., Puchhammer-Stöckl E., Weseslindtner L., Zoufaly A., Aberle J.H., Stiasny K. (2022). Heterogeneous SARS-CoV-2-Neutralizing Activities after Infection and Vaccination. Front. Immunol..

[45] Khaleeq S., Sengupta N., Kumar S., Patel U.R., Rajmani R.S., Reddy P., Pandey S., Singh R., Dutta S., Ringe R.P. (2023). Neutralizing Efficacy of Encapsulin Nanoparticles against SARS-CoV2 Variants of Concern. Viruses.

[46] Dhawan M., Saied A.R.A., Mitra S., Alhumaydhi F.A., Emran T.B., Wilairatana P. (2022). Omicron Variant (B.1.1.529) and Its Sublineages: What Do We Know so Far amid the Emergence of Recombinant Variants of SARS-CoV-2?. Biomed. Pharmacother..

[47] Wietschel K.A., Fechtner K., Antileo E., Abdurrahman G., Drechsler C.A., Makuvise M.K., Rose R., Voß M., Krumbholz A., Michalik S. (2024). Non-Cross-Reactive Epitopes Dominate the Humoral Immune Response to COVID-19 Vaccination—Kinetics of Plasma Antibodies, Plasmablasts and Memory B Cells. Front. Immunol..

